# Barriers and enablers for the implementation of trauma-informed care in healthcare settings: a systematic review

**DOI:** 10.1186/s43058-023-00428-0

**Published:** 2023-05-05

**Authors:** Yan Huo, Leah Couzner, Tim Windsor, Kate Laver, Nadeeka N. Dissanayaka, Monica Cations

**Affiliations:** 1 College of Education, Psychology and Social Work, Flinders University, Adelaide, SA Australia; 2 College of Medicine and Public Health, Flinders University, Adelaide, SA Australia; 3 UQ Centre for Clinical Research, Faculty of Medicine, University of Queensland, Brisbane, QLD Australia; 4 School of Psychology, The University of Queensland, Brisbane, QLD Australia; 5 Department of Neurology, Royal Brisbane and Woman’s Hospital, Brisbane, QLD Australia

**Keywords:** Trauma-informed care, Healthcare, Health services, Psychological trauma, Posttraumatic stress disorder, Care quality and safety

## Abstract

**Background:**

Healthcare services can be re-traumatising for trauma survivors where they trigger memories of past distressing events and exert limits to a survivor’s sense of autonomy, choice, and control. The benefits of receiving trauma-informed healthcare are well established; however, factors that promote or impede the implementation of trauma-informed care are not yet well characterised and understood.

The aim of this review was to systematically identify and synthesise evidence regarding factors that promote or reduce the implementation of TIC in healthcare settings.

**Methods:**

This systematic review followed the Preferred Reporting Items for Systematic Reviews and Meta-analyses (PRISMA) 2.0 guidelines. Scopus, MEDLINE, Proquest, PsycINFO and grey literature were searched for original research or evaluations published between January 2000 and April 2021 reporting barriers and/or facilitating factors for the implementation of trauma-informed care in a healthcare setting. Two reviewers independently assessed the quality of each included study using the Mixed Methods Appraisal Tool (MMAT) Checklist.

**Results:**

Twenty-seven studies were included, 22 of which were published in the USA. Implementation occurred in a range of health settings, predominantly mental health services. The barriers and facilitators of implementing trauma-informed care were categorised as follows: intervention characteristics (perceived relevance of trauma-informed care to the health setting and target population), influences external to the organisation (e.g. interagency collaboration or the actions of other agencies) and influences within the organisation in which implementation occurred (e.g. leadership engagement, financial and staffing resources and policy and procedure changes that promote flexibility in protocols). Other factors related to the implementation processes (e.g. flexible and accessible training, service user feedback and the collection and review of initiative outcomes) and finally the characteristics of individuals within the service or system such as a resistance to change.

**Conclusions:**

This review identifies key factors that should be targeted to promote trauma-informed care implementation. Continued research will be helpful for characterising what trauma-informed care looks like when it is delivered well, and providing validated frameworks to promote organisational uptake for the benefit of trauma survivors.

**Registration:**

The protocol for this review was registered on the PROSPERO database (CRD42021242891).

**Supplementary Information:**

The online version contains supplementary material available at 10.1186/s43058-023-00428-0.

## Contributions to the literature

- The impacts of psychological trauma have important implications for the provision and receipt of healthcare.
- This systematic review identifies key factors that should be targeted to promote trauma-informed care implementation, including interagency collaboration, staff and leadership buy-in, aligning implementation strategies with existing policies and procedures, allocation of adequate human and financial resources, flexibility in organisational policies and procedures, ongoing and tailored training, participatory co-design, and the collection and monitoring of data.
- Identifying factors that influence implementation success across trauma-informed care initiatives can help to inform the selection of implementation strategies and planning.

## Background

Up to 70% of the population will experience exposure to one or more psychologically traumatic event in their lifetime [1]. Psychologically traumatic events are those events perceived by the individual as threatening to their safety and/or overwhelming to their ability to understand and make sense of the experience [2]. While most people will recover from traumatic stress, exposure to these events can have lasting adverse effects including reduced quality of life and risk for psychological disorders, non-suicidal self-injury, and suicide [3, 4].

The impacts of psychological trauma on interpersonal skills, perception, problem-solving, and other core abilities and experiences have important implications for the provision and receipt of healthcare. Healthcare services can present risk for re-traumatisation where they trigger memories of past distressing events and exert limits to a survivor’s sense of autonomy, choice, and control [5, 6]. Hypervigilance to threat and impaired emotion regulation skills mean that care behaviours and environments can trigger a fight or flight response that can manifest as externalising (e.g. aggression) or internalising behaviour (e.g. withdrawal). This can result in the use of seclusion and restraint, which have major negative impacts on quality of life and quality of healthcare services [7].

Recognition of these impacts prompted the development of trauma-informed care in mental health and other settings where most service users have experienced psychological trauma [7–9]. Trauma-informed care (TIC) is a care approach in which services are organised to ensure that all staff have a basic understanding of the potential impact of traumatic stress and can amend care to promote safety, choice, autonomy, collaboration, and respect. Staff in TIC settings are not necessarily expected to treat the symptoms of trauma, but pathways for care recipients to access treatments for trauma are known and used by all staff [10].

Organisational interventions aiming to promote the delivery of trauma-informed care have been implemented in healthcare settings in several contexts, including mental health services, inpatient emergency departments, hospital wards, and palliative care [10, 11]. For the purposes of this review, trauma-informed organisational interventions refer to organisation-level interventions (as opposed to individual clinician or service user interventions) aiming to improve staff awareness and understanding of traumatic stress across an organisation (or within a specific group of staff), and/or establishing organisational policies and processes to meet trauma-related needs. Efforts to improve the capability of clinicians to deliver trauma therapies and treatments are out of scope of this review.

Research has demonstrated that organisational interventions to promote delivery of trauma-informed healthcare can promote well-being among survivors, improve staff skills and collaboration, reduce the use of seclusion and restraint, and reduce the prevalence of the secondary effects of trauma including drug and alcohol use [6]. However, the implementation of TIC involves a paradigm shift that requires a complex organisational change process encompassing workforce upskilling, organisational change, development of clear referral pathways, environmental change, and other implementation strategies [12, 13]. Such broad change requires significant time and resources, and evaluation of outcomes at the organisational and/or systems level. Factors that promote or impede implementation of TIC are not yet well characterised and understood [10]. Understanding contextual, organisational, and implementation-specific factors that promote the uptake and effectiveness of an intervention can help to guide more efficient and sustainable implementation. This review, which sits on the ‘green line’ of the implementation science subway [14], is a critical step toward identifying and designing effective implementation strategies to implement TIC more widely.

As such, the aim of this review was to systematically identify and synthesise evidence regarding factors that promote or reduce the effectiveness and/or implementation of TIC in healthcare settings.

The research questions to be answered were:

- What facilitating factors improve the effectiveness and/or implementation of TIC in healthcare settings?
- What factors act as barriers that limit the effectiveness and/or implementation of TIC in healthcare settings?

## Methods

We conducted a systematic review following the guidance of the Cochrane Qualitative and Implementation Methods Group Guidance Series and report our findings according to the Preferred Reporting Items for Systematic Reviews and Meta-Analyses 2.0 (PRISMA 2.0) guidelines [15]. A checklist of PRISMA 2.0 items is presented in Supplementary Table S1. The review protocol was registered on the PROSPERO database (CRD42021242891).

### Data sources and searches

We conducted a search of Scopus, MEDLINE, Proquest, and PsycINFO for English language studies published from January 2000 to April 2021. The search strategy is presented in Supplementary Table S2 and combined concepts related to the intervention (TIC), the setting (healthcare settings), and the outcome (barriers and facilitating factors for implementation). Reference lists of all included studies were hand-searched for additional records. We also searched grey literature via a Google search, Open Grey Europe, the Grey Literature Report, Web of Science, and report publications from relevant peak bodies (e.g. the International Society for Traumatic Stress Studies, American Psychiatric Association, Phoenix Australia).

### Eligibility criteria

#### Study type

We included studies published since the year 2000 and in English, reporting original research or evaluation, and that reporting barriers and/or facilitating factors for the effectiveness or implementation of a TIC initiative in a healthcare setting. Studies published prior to 2000 were excluded because the TIC framework was not well-defined in research before this time [8]. Studies were excluded if they did not report original research or evaluation data (e.g. literature reviews, study protocols), were published prior to the year 2000, could not be accessed by the research team, or were not available in English.

#### Intervention and implementation strategies

Studies included in this review described strategies to implement TIC. TIC has been criticised for lacking an agreed operational definition [16] and what constitutes ‘trauma-informed care’ varies between settings. For this review, interventions or systems of care specifically described as “trauma-informed” were included. Similarly to previous reviews on TIC (e.g. [17]), we were deliberately broad in accepting the authors’ definition of TIC given the lack of an existing operational definition. Broadly, we accepted studies that described efforts to improve staff awareness and understanding of psychological trauma and organisational policies and processes to better to meet trauma-related needs.

Included studies described implementation at the organisational level. In this case, ‘initiative’ is used to describe a discrete strategy or set of strategies aiming to implement the principles of TIC within the organisation. An organisational initiative was defined as any initiative listed in the Cochrane Effective Practice and Organisation of Care Review Group data collection taxonomy [18] Sects. 2.1.3 (e.g. revision of professional roles, environmental changes, consumer participation in governance) or educational strategies listed in 2.1.1 (e.g. distribution of educational materials, local opinion leaders) as long as they were conducted at an organisation level. That is, educational strategies had to be delivered within an organisation/system to all staff or key staff who were expected to diffuse the information to others. System-level interventions to improve access or pathways to suitable trauma treatments were included.

Studies describing interventions targeted at individual clinicians (e.g. professional development to deliver a particular therapy for PTSD) or individual service users (e.g. evaluating the effectiveness of particular PTSD treatments) were excluded.

#### Setting

‘Healthcare organisations’ included primary, secondary and tertiary healthcare settings (e.g. acute and subacute hospital services, primary care, outpatient clinics, residential mental health treatment centres, ambulatory care, etc.). Mental health services, including drug and alcohol services, were included. Child welfare and out-of-home care services (including residential welfare centres) were excluded unless they included a healthcare component.

#### Outcomes

Finally, included studies were required to measure and report quantitative and/or qualitative data regarding factors that influenced initiative or implementation outcomes. Barriers were defined as any factor reported to impede implementation efforts, and facilitators were any factors reported to enable implementation.

### Study screening and data extraction

One reviewer (YH) screened all titles and removed irrelevant papers. Abstracts and full texts were screened for eligibility by two reviewers (YH and MC) using an eligibility checklist based on the criteria described above. Disagreements about inclusion were resolved via discussion between the reviewers, and a third reviewer (LC) was consulted where consensus could not be reached. Study authors were contacted where more information was required to confirm eligibility for inclusion in this review. Data extraction was conducted by one reviewer (YH) using a data extraction spreadsheet that was piloted with five studies before being finalised and being used with the remaining studies. The accuracy of data extraction was validated by a second reviewer. Extracted data included the study’s first author and year of publication, design, setting, population, number of sites, initiative elements (e.g. implementation strategies), outcome data type (e.g. qualitative, quantitative, mixed), evaluation method, implementation outcomes reported, barriers identified, and facilitating factors identified. Intervention outcomes were out of scope of this review so were not reported here.

### Quality assessment

The Mixed Methods Appraisal Tool (MMAT) Checklist, shown in Supplementary Table S3, was used to assess the quality of each included study. The validity, robustness, and applicability of each included study was appraised by two team members (YH and BW-H) independently and in duplicate [19].

### Data management, analysis, and synthesis

The implementation strategies used in each study were synthesised into broad categories and mapped to the ERIC compilation of implementation strategies independently by two authors (YH and MC). These authors examined the features of each strategy and aligned these features with the most closely related category, and disagreements (which occurred for 8 out of 27 studies) were resolved on discussion with a third reviewer (LC).

The core aim of this paper was the synthesise data about barriers and facilitating factors for implementation. As such, this process involved a more detailed, two-step process modelled on the method of a recent review of barriers and facilitating factors for person-centred care in long-term care settings [20]. First, two authors (YH and MC) independently used a thematic analysis approach to group barriers and facilitating factors into recurrent themes (e.g. lack of collaboration, time constraints). Themes were consolidated on discussion and a second independent round of coding was conducted by both reviewers with new emerging themes added to the codebook iteratively. A final, third round of coding was conducted by one reviewer for refinement (MC). In the second step of the synthesis, the themes were mapped to the Consolidated Framework for Implementation Research (CFIR) by both reviewers [21]. All discrepancies were resolved via discussion. Mapping to CFIR aimed to provide organised guidance to researchers, service providers, and policy makers about the key contextual and initiative features that promote or limit success when implementing TIC. CFIR is a determinant framework designed to predict or explain barriers and facilitators to implementation success [22], and is therefore well suited to our aim of capturing, organising and describing common barriers and facilitators to implementing TIC organisational interventions.

## Results

The initial search identified 3051 original results, of which 170 were retrieved in full text and screened against the review inclusion and exclusion criteria. Most exclusions were due to the implementation occurring in a non-health-related care setting. A total of 27 studies were included, reported across 28 publications (Supplementary Figure S1).

### Study characteristics and implementation strategies

Characteristics of the included studies are described in Table 1. Of the 27 included studies, 22 described efforts to implement TIC into healthcare settings in the United States of America (USA). The remaining five studies were conducted in Australia (*n* = 3) and Canada (*n* = 2). Implementation occurred in mental health settings (*n* = 15), maternal and women’s health settings (*n* = 2), paediatric health settings (*n* = 2), primary care clinics (*n* = 3), emergency departments (*n* = 1), or across whole systems within a geographical area including health, policy, child welfare, and other social services (*n* = 4). Implementation occurred within single health sites for eight studies, and the remainder reported implementation in multiple sites or across a whole service system. Nine of the studies explicitly described using existing implementation frameworks, theories and/or models to design and evaluate their implementation strategies, including the Exploration, Preparation, Implementation and Sustainment (EPIS) framework [23, 24], the Service Integration Framework [25, 26], Chen’s theoretical framework for program evaluation [27, 28], CFIR [21, 29], and models of rapid cycle implementation [30, 31]. Other studies conducted a review of TIC implementation literature but did not explicitly design their strategy or evaluation against a specific framework (e.g. [32, 33]).

**Table 1:** Characteristics of included studies

Study (country)	Study design	Study setting	Population	# of sites	Implementation strategies	Outcome data type
Amaro, 2005 [25] (USA)	Case study	Drug and alcohol, residential, outpatient and inpatient	Adult women	5	Staff education (e.g. staff education, manualised intervention protocols) Organisational strategies (e.g. project steering committee, establishing a shared philosophy, shared resources, routine trauma screening) Consumer-orientated strategies (e.g. group therapy, peer support group)	Qual
Azeem, 2015 [34] (USA)	Case study	Psychiatric hospital, inpatient	Children	1	Staff education Organisational interventions (e.g. executive support and consultation, audit and feedback, routine screening, restraint reduction and prevention tools, debriefing) Consumer-orientated strategies (e.g. consumer involvement, calming and recreational activities)	Mixed
Bartlett, 2016 [35] (USA)	Case study, Single group pre-post	Child welfare area offices and mental health network, community	Children	20	Staff education and communication (e.g. staff training, staff wellness classes) Organisational interventions (e.g. development of trauma-informed leadership teams, monthly planning meetings) Consumer-orientated strategies: (e.g. statewide implementation of three evidence-based trauma treatments using learning collaboratives, creating a welcoming space for children and families, trauma workshops for resource (foster) parents, local schools, and community providers)	Mixed
Beidas, 2016 [23] (USA)	Case study	Behavioural health organisations, inpatient, residential, outpatient, and community	Children and adolescents	> 14	Staff education and communication (e.g. staff training, consultation and technical assistance) Organisational strategies: (e.g. routine screening, cross-organisational collaboration, building a referral network) Consumer-orientated strategies (e.g. implementation of trauma-focussed therapies)	Quant
Caldwell, 2014 [36] (USA)	Case study	Psychiatric hospital, inpatient	Children	1^b^	Staff education and communication (e.g. staff supervision, new employee orientation) Organisational strategies (e.g. strategic plan, leadership support and weekly meetings, policy and procedure change, individualised treatment and safety plans, routine debriefing) Consumer-orientated strategies (e.g. family education, child and family inclusion in implementation)	Mixed
Chandler, 2008 [37] (USA)	Cross-sectional	Psychiatric hospital, inpatient	Adult	1	Staff education (e.g. staff training) Organisational strategies (e.g. patient management plan, patient safety checklist, shared resources)	Qual
Conover, 2015 [38] (USA)	Case series, Single group pre-post	Behavioural health organisations, setting NR	NR	32	Staff education (e.g. staff education and supervision, learning community) Organisational strategies (e.g. establishing an implementation team, organisational assessment, offering trauma-specific evidence-based practice)	Mixed
Damian, 2017 [39] (USA)	Single-group pre-post	Police, social services, health, and education agencies, setting NR	All	90^a^	Staff education (e.g. monthly staff training, ongoing coaching and feedback) Organisational strategies (e.g. implementation of trauma-informed care policies and practices)	Mixed
Dike, 2020 [40] (USA)	Interrupted time series	Psychiatric hospital, inpatient	Adult	27	Staff education (e.g. staff training, recognition of good performance) Organisational strategies: (e.g. project steering committees, routine screening, comfort rooms, policy and procedure change, behavioural consultation service, audit and feedback, patient participation, debriefing protocols,) Consumer-orientated strategies (e.g. sensory modulation interventions)	Quant
Dorr, 2019 [27] (USA)	Case study	Psychiatric hospital, inpatient	Adolescent	1	Staff education (e.g. staff training and supervision, role play) Organisational strategies (e.g. case review protocols, new staff hiring protocols, collaborative patient-staff problem-solving assessment process, environmental change, nominated program implementers) Consumer-orientated strategies (e.g. daily programming schedule which allowed patient-staff interaction)	Mixed
Dueweke, 2019 [41] (USA)	Single group pre-post	Primary care, community	Children	1	Staff education Organisational strategies (e.g. availability of routine screening tools)	Mixed
Hale, 2020 [42] (USA)	Case study	Psychiatric hospital, inpatient	Children and adolescents	3	Staff education (e.g. culture change, staff training and debriefing) Organisational strategies (e.g. updated policies and procedures, de-escalation techniques, case review) Consumer-orientated strategies (patient education)	Mixed
Hall, 2016 [43] (Australia)	Single-group pre-post	Emergency departments, inpatient	All	2	Staff education (staff training)	Mixed
Huntington, 2005 and Moses 2003 [44, 45] (USA)	Case series	Drug and alcohol, mental health, public health, and education services, residential, outpatient and community	Adult women	9	Staff education and communication (e.g. monthly-quarterly group meetings, staff training) Organisational strategies (e.g. interagency steering committee, formal voting mechanism to resolve issues) Consumer-orientated strategies (e.g. trauma-specific services, peer-run services, consumer involvement, trauma consultation-liaison services)	Qual
Jee, 2020 [46] (USA)	Single group pre-post	Primary care, community	Children	1	Staff education (e.g. staff training)	Mixed
Korchmaros, 2021 [33] (USA)	Single group longitudinal	Drug and alcohol, residential	Adolescent	1	Staff education (e.g. staff training) Organisational strategies (e.g. steering group, agency-specific protocols, routine safety plans and red flag reviews) Consumer-orientated strategies (e.g. client-specific Seeking Safety groups)	Quant
Koury, 2017 [47] (USA)	Case study	Drug and alcohol, setting NR	Adolescent	30	Staff education (e.g. learning collaborative, training ‘champions’, monthly consultations, resource sharing)	Qual
Levine, 2021 [48] (Canada)	Cross-sectional	Primary care clinics, community	All	4	Staff education (e.g. interprofessional education sessions, group discussions)	Qual
Loomis, 2019 [32] (USA)	Case study	Whole of city health system, all settings	All	City-wide	Staff education (e.g. learning collaborative, workforce training, monthly meetings, train-the-trainer program) Organisational strategies (e.g. leadership engagement, expert workgroup available to guide implementation, resources to promote policy and procedure change)	Qual
Mantler, 2018 [49] (Canada)	Case series	Health services delivered within domestic violence services, community	Adult women	3	Organisational strategies (e.g. integration of nurse practitioner clinic within domestic violence services)	Qual
McEvedy, 2017 [50] (Australia)	Cross-sectional	Mental health, all settings	All	19	Staff education (e.g. train-the-trainer program)	Qual
McNamara, 2021 [51] (USA)	Single group pre-post	Paediatric hospital, inpatient	Children	2	Staff education (e.g. staff training)	Quant
Palfrey, 2019 [52] (Australia)	Single group pre-post	Mental health, all settings	All	102^a^	Staff education (e.g. staff training, regular supervision) Organisational strategies (e.g.routine screening)	Mixed
Purbeck, 2020 [29] (USA)	Case series	Mental health and drug and alcohol, community, outpatient and residential	Children	7	Staff education (e.g. staff training and consultation, monthly team meetings) Organisational strategies (e.g. fostering practice-based capacities, assessing readiness, capacity building) Consumer-orientated strategies (e.g. engaging children and caregivers)	Mixed
Sala-Hamrick, 2021 [30] (USA)	Cross-sectional	Primary care, community	Children	1	Staff education (e.g. staff training, collaborative creation of implementation strategy) Organisational strategies (e.g. routine screening, shared resources, data availability)	Mixed
Simonich, 2015 [53] (USA)	Single group pre-post	All childhood services across state, system	Children	State-wide	Staff education (e.g. staff training, train-the-trainer program, annual clinician meetings) Organisational strategies (e.g. established network of trained clinicians))	Mixed
Tuck, 2017 [54] (USA)	Case study	Maternal health services, community	Adult women	City-wide	Staff education (e.g. staff training, coaching) Organisational strategies (e.g. working group, policy and procedure change)	Mixed

*Abbreviations*: *NR* not reported, *USA* United States of America

^a^Number of participants, paper did not report number of sites; ^b^ Only the health setting was included here

Strategies used to implement the principles of TIC were similar across the included studies. Mapped to the ERIC compilation of implementation strategies [55], all but one study included some form of staff education and training, ranging from a single educational meeting (e.g. [41, 43]), implementing train-the-trainer strategies [32, 50, 53], providing regular clinical supervision [27, 36, 38, 52], creating learning collaboratives [32, 47], modelling change [37, 40, 41, 34], developing educational materials for new employees [32, 42, 48, 34], to conducting ongoing training (e.g. [39]). Some programs identified and prepared ‘champions’, who received (or had pre-existing) a higher level of training and were available as peer mentors [47, 34], while others committed resources to the ongoing availability of experts for consultation [25, 35–37, 44, 45].

Most studies paired staff training and education with other implementation strategies to embed TIC throughout services. Several initiatives included activities to build buy-in and a shared rationale for implementing TIC within the organisation, including by developing position statements (e.g. [25]), aligning strategic planning with the TIC principles (e.g. [54, 34]), conducting team building exercises (e.g. [44, 45]), and establishing written agreements between participating agencies (e.g. [54]). Several initiatives chose to establish a team of staff responsible for implementation and monitoring [29, 35, 38, 42] while others elected a single staff member to drive and oversee implementation (e.g. [27]). Other common implementation strategies included organising quality monitoring systems, including increasing the availability and/or routine use of screening for trauma-related needs [23, 25, 30, 40, 41, 44, 45, 52], education outreach activities to other agencies [23, 44, 45], mandating change via policy and procedure change [32, 33, 36, 39, 40, 42, 54, 34], and clinical team group debriefing and care planning after critical incidents [36, 40, 42, 44, 45, 47, 34].

### Evaluation methods and quality appraisal

Most of the included studies examined barriers and facilitating factors for implementation of TIC using mixed-methods including staff interviews and/or focus groups, process data (e.g. uptake of screening tools and training attendance), and outcome data (e.g. rates of seclusion and restraint use). Eight studies reported across nine papers described author reflections on barriers and facilitating factors for implementation rather than reporting formal data [27, 32, 36, 38, 40, 42, 44, 45, 34]. This approach is subject to a high risk of bias, as assessed using the MMAT (see Supplementary Table S4 for full methodological quality appraisal). The methodological quality of the other included studies was moderate, with most reporting clear research questions, well-justified data collection methods, and representative data collection from a diverse group of staff and/or service users. Common methodological limitations included that few of the studies reporting quantitative data considered the impact of confounding factors in their analysis, and the mixed-methods studies rarely attempted or described their approach to data integration. Importantly, none of the included studies explicitly compared implementation strategies to each other and so could not identify which were more effective than others.

### Barriers and facilitating factors for implementation

Barriers and facilitating factors for implementing TIC across the included studies are described in Table 2 and mapped to the CFIR framework in Table 3.

**Table 2:** Barriers and facilitating factors for implementation identified in included studies

Study (country)	Evaluation method ^a^	Outcomes reported ^b^	Barriers identified	Facilitators identified
Amaro, 2005 [25] (USA)	Field notes Focus group	Acceptability Effectiveness (patient satisfaction)	Staff resistance to change Lack of communication and collaboration Lack of consumer engagement Reliance on volunteers Insufficient training Insufficient staff skill mix Lack of data collection and evaluation Staff time constraints	Leadership buy-in Ongoing staff training Ongoing availability of experts Intervention tailored for culturally and linguistically diverse groups
Azeem, 2015 [34] (USA)	File audit Field notes	Adoption Feasibility Effectiveness (restraint use)	NR	Staff buy-in Leadership buy-in TIC embedded into strategic planning Policy and procedure change Data collection and evaluation Ongoing availability of experts Collaboration and teamwork Consumer involvement Regular debriefing
Bartlett, 2016 [35] (USA)	Survey Interviews Focus group File audit Field notes	Adoption Feasibility Sustainability Effectiveness (psychiatric symptoms and behaviour problems)	Staff time constraints Staff turnover Competing priorities Lack of appropriate services Lack of leadership commitment Financial constraints Lack of knowledge about steps for implementing TIC	Ongoing availability of experts Events that promote collaboration
Beidas, 2016 [23] (USA)	Survey File audit Field notes	Acceptability Feasibility Adoption Penetration	Staff turnover Lack of perceived relevance of TIC Staff time constraints Staff resistance to change Inflexible policies and procedures	Leadership buy-in Financial incentives Resources allocated to staff who can coordinate collaboration between staff and organisations Availability of structured screening tools Community and academic partnership
Caldwell, 2014 [36] (USA) ^c^	File audit Field notes	Adoption Effectiveness (restraint/seclusion usage)	NR	Leadership buy-in Data collection and monitoring Consumer engagement Ongoing training for all staff and availability of experts Regular debriefing
Chandler, 2008 [37] (USA)	Interviews	Acceptability Adoption Penetration Feasibility Sustainability	Restricted budget Confined physical space	Culture of support, respect for staff Flexibility in policies and procedures Ongoing availability of experts Flexible protocols Modelling Adequate staffing ratios
Conover, 2015 [38] USA	File audit Field notes	Adoption	Lack of data collection and monitoring	NR
Damian, 2017 [39] USA	Survey Interview	Acceptability Adoption	NR	Policy and procedure change Provision of appropriate physical space and design Staff appreciation from leadership Prioritisation of staff self-care
Dike, 2020 [40] USA	File audit Field notes	Adoption Acceptability Costs Effectiveness (restraint use and staff injury)	Staff resistance to change Staff time constraints Competing priorities	Leadership buy-in Modelling Consumer involvement Having a precedent established by earlier implementation efforts in other states Data collection and monitoring On-site psychologists Financial resources
Dorr, 2019 [27] USA	File audit Field notes	Adoption Acceptability Sustainability Effectiveness (restraint/seclusion usage)	Insufficient training Staff resistance to change Lack of ongoing training or training for new staff Lack of collaboration and communication within organisation Lack of multidisciplinary teamwork Limited ongoing feedback and evaluation Low perceived relevance of TIC Lack of trust in leadership Lack of staff confidence to implement TIC Insufficient preparation Uneven allocation of financial resources	Provision of appropriate physical space and design Recruitment of staff open to TIC implementation Culture that valued evidence-based practice and high-quality care Financial resources
Dueweke, 2019 [41] USA	File audit Interviews Surveys	Acceptability Adoption Feasibility Penetration	Staff time constraints Lack of collaboration and communication within organisation Fear of retraumatising clients Screening tools too long for use in clinical practice	Perceived relevance of TIC Ongoing availability of resources Modelling Interactive, clear, and straightforward training approach Availability of structured screening tools
Hale, 2020 [42]USA	File audit Field notes	Acceptability Adoption Sustainability Effectiveness (restraint/seclusion use)	NR	Mapping TIC to existing organisational priorities Leadership buy-in Implementation group with representation from staff at all levels Data collection and monitoring Regular debriefing Policy and procedure change Targets and incentives Training included in new employee orientation and yearly competencies
Hall, 2016 [43] Australia	Survey Focus groups Field notes	Acceptability Adoption Penetration Feasibility	Staff time constraints Staff resistance to change	Involvement of person with lived experience of trauma as training co-facilitator
Huntington, 2005 USA, and Moses, 2003 [44, 45] USA	File audit Field notes	Acceptability Adoption Fidelity Feasibility Sustainability	Staff resistance to change Staff time constraints Staff turnover Competing priorities Financial constraints Lack of interagency collaboration and communication	Activities to promote integration including team building and developing shared philosophy Consumer involvement Flexibility and training to promote consumer involvement Ongoing availability of experts Partnerships with referring and support organisations Reduce use of jargon Mentoring and supervision Leadership and stakeholder buy-in Outreach to promote TIC in other organisations Availability of structured screening tools Resources allocated to staff who can coordinate collaboration between staff and organisations Interagency committees Strategies to increase training accessibility Perceived relevance of TIC Policy and procedure change Regular debriefing
Jee, 2020 [46] USA	Survey Focus group Interviews	Acceptability Adoption Feasibility	Staff time constraints Additional training required Lack of debriefing Competing priorities	Use of videos within training
Korchmaros, 2021 [33] USA	Survey	Fidelity Feasibility Acceptability Sustainability	Commitment to TIC by leadership not sustained over time Low staff confidence in ability to implement TIC	Policy and procedure change
Koury, 2017 [47] USA	File audit Field notes Survey	Acceptability Feasibility	Lack of access to technology and technological difficulties Staff time constraints	Regular debriefing Staff accountability Homework and monthly consultations following training Participants supporting each other and having a sense of being a team
Levine, 2021 [48] Canada	Field notes Interviews	Acceptability Adoption Feasibility	Staff resistance to change Competing priorities Lack of staff teamwork Financial constraints Staff time constraints Policy and regulation not consistent with TIC Lack of knowledge about steps for implementing TIC Systemic racism	Perceived relevance of TIC Embedding TIC into new staff orientation A supportive, flexible work environment Other TIC initiatives in the community Ongoing interprofessional discussions about trauma after training
Loomis, 2019 [32] USA	File audit Field notes	Feasibility Fidelity	Financial constraints Staff time constraints Competing priorities Staff turnover	Intervention development included workforce feedback Leadership engagement Training delivered to all levels of organisational hierarchy Regular TIC training across multiple sites TIC training incorporated into employee orientation Embedding TIC principles into existing initiatives; no additional initiatives
Mantler, 2018 [49] Canada	Interviews Field notes Survey Field notes	Acceptability Feasibility Fidelity Sustainability Effectiveness (patient satisfaction)	Financial constraints Lack of external services providing TIC Lack of trust in healthcare providers Staff time constraints Lack of interagency collaboration and communication:	Integration and co-location of health and domestic violence services Policy and procedure change Collaboration between service providers
McEvedy, 2017 [50] Australia	Interviews Focus group	Acceptability Adoption Appropriateness Penetration	Lack of organisational support Staff resistance to change Competing priorities Staff not strategically selected for training Training content not practical enough Training too long	Tailored training Making the training compulsory and rostered staff to attend Targeting experienced educators for train-the-trainer training Establishing a multidisciplinary team including consumers Staff openness to change
McNamara, 2021 [51] USA	Survey File audit	Acceptability Adoption Penetration	Staff turnover Competing priorities TIC training voluntary, poor uptake	Consumer engagement Conducting workshops in a variety of educational environments
Palfrey, 2019 [52] Australia	Survey Focus group	Acceptability Adoption Feasibility Penetration Sustainability	NR	Perceived relevance of TIC Practical components of training (e.g. role plays) Training content about trauma presentation, neurobiology, and prevalence
Purbeck, 2020 [29] USA	Survey Focus group Interviews	Acceptability Appropriateness Adoption Feasibility	Low perceived relevance of TIC Program complexity Staff time constraints	Internal implementation leaders with dedicated time for implementation Ongoing availability of experts Regular meetings between clinical team and evaluation team Having a full-time evaluator Staff time dedicated to implementation Clinical supervision that integrated the initiative Sessions with clients are long enough to implement the intervention Staff openness to change
Sala-Hamrick, 2021 [30] USA	File audit Focus group	Acceptability Adoption Feasibility Fidelity Penetration	Staff time constraints Staff lack of confidence	Data collection and evaluation Consistent use of trauma screening Visual screening reminders Availability of structured screening tools Strengths-based focus Adequate preparation before implementation Ongoing training Staff given time to develop their confidence
Simonich, 2015 [53] USA	Field notes Survey	Feasibility Fidelity	Lack of routine trauma screening Lack of skill in trauma identification among referring services Lack of awareness of TIC among referring services	Engaging and training other relevant child-serving systems and referring services Flexible training curriculum
Tuck, 2017 [54] USA	Focus groups Survey	Feasibility Fidelity Effectiveness (participant experience)	Lack of data collection and monitoring	Leadership buy-in Interagency collaboration Financial resources Prior familiarity with TIC

*Abbreviations*: *TIC* trauma-informed care, *NR* not reported, *USA* United States of America

^a^‘Field notes’ includes observations of clinical and educational practices, and author reflections; ‘file audit’ includes review of case notes and organisational records, and organisational self-assessment

^b^‘Acceptability’ includes staff attitudes toward and confidence with TIC; ‘feasibility’ includes staff knowledge and skill with delivering TIC; ‘adoption’ includes organisational change

^c^Only the health setting included here

**Table 3:** Barriers and facilitating factors for implementation mapped to Consolidated Framework for Implementation Research

**Study (country)**	Intervention characteristics	Outer setting	Inner setting	Characteristics of individuals	Process
Amaro, 2005 [25] (USA)			✓	✓	✓
Azeem, 2015 [34] (USA)			✓		✓
Bartlett, 2016 [35] (USA)			✓		✓
Beidas, 2016 [23] (USA)	✓	✓	✓	✓	✓
Caldwell, 2014 [36] (USA)			✓		✓
Chandler, 2008 [37] (USA)			✓		✓
Conover, 2015 [38] (USA)					✓
Damian, 2017 [39] (USA)			✓		✓
Dike, 2020 [40] (USA)		✓	✓	✓	✓
Dorr, 2019 [27] (USA)	✓		✓	✓	✓
Dueweke, 2019 [41] (USA)	✓		✓		✓
Hale, 2020 [42] (USA)			✓		✓
Hall, 2016 [43] (Australia)			✓		✓
Huntington, 2005, and Moses, 2003 [44, 45] (USA)	✓	✓	✓	✓	✓
Jee, 2020 [46] (USA)			✓		✓
Korchmaros, 2021 [33] (USA)			✓		✓
Koury, 2017 [47] (USA)			✓		✓
Levine, 2021 [48] (Canada)	✓	✓	✓	✓	✓
Loomis, 2019 [32] (USA)			✓		✓
Mantler, 2018 [49] (Canada)		✓	✓		
McEvedy, 2017 [50] (Australia)			✓	✓	✓
McNamara, 2021 [51] (USA)			✓		✓
Palfrey, 2019 [52] (Australia)	✓				✓
Purbeck, 2020 [29] (USA)	✓		✓	✓	✓
Sala-Hamrick, 2021 [30] (USA)			✓	✓	✓
Simonich, 2015 [53] (USA)		✓	✓		✓
Tuck, 2017 [54] (USA)		✓	✓		✓

*Abbreviations*: *USA* United States of America

#### Intervention characteristics

Characteristics of the intervention (TIC) were reported to influence implementation across seven studies, always related to the perceived relevance of TIC to the setting and their target population. Three studies reported that staff did not perceive that the principles of TIC were suitable for their organisation or that their service users were too diverse to make delivery of TIC possible [23, 27, 29]. Four other studies reported that a high level of perceived relevance of TIC among staff was a facilitating factor for implementation in their sites [41, 44, 48, 52].

#### Outer setting

Seven studies reported barriers and facilitating factors associated with the outer setting (that is, influences external to the organisation itself). One study noted that other services implementing TIC set a precedent and created a sense of peer pressure for the organisation to also pursue implementation [40]. A culture of interagency collaboration was noted as a facilitating factor in some studies, particularly where funding was allocated for administrative support to coordinate and monitor the collaboration [44, 45, 53, 54]. Simonich et al. [53], Huntington et al. [44] and Mantler et al. [49] all noted that the implementation of TIC in other agencies servicing their clients was important to implementation success in their own organisation. That is, even where clients received TIC services from their organisation, this was undermined by other agencies delivering care that reduced client trust and sense of safety with healthcare providers. Outreach and training to other organisations was a facilitating factor for implementation in two of these studies [44, 53]. Broader policy, funding arrangements, and regulation (external to the organisation) that was not consistent with the delivery of TIC was noted as a barrier to implementation in one study [48].

#### Inner setting

Factors related to the inner setting (the organisation or system in which implementation occurred) were reported as barriers or facilitating factors for implementation across 25 of the 27 studies. In many cases, this referred to the culture of the organisation and climate for implementation. Common facilitating factors included high levels of engagement and commitment from organisational leadership [23, 25, 32, 36, 40, 42, 44, 45, 54, 34], the alignment of TIC with existing organisation strategic plans or policies [32, 42, 34], a culture of support for staff and evidence-based practice (including giving staff adequate time to learn and adopt new practices) [27, 30, 39], and allocation of adequate financial and staffing resources to promote implementation [23, 27, 40, 54]. Where financial resources were not allocated to the initiative, or these were insufficient, this was a barrier to implementation [32, 35, 44, 47–49]. One study also noted that although the provision of financial resources was a facilitating factor, the uneven distribution of these resources (favouring changes to the physical environment over investment in staff and human resources) was a barrier to change [27].

Other common barriers to implementation included competing priorities and staff time constraints [23, 25, 29, 30, 32, 35, 40, 41, 43–48], a lack of collaboration between teams within the organisation [27], and policies that were incompatible with delivering TIC. In particular, organisational policies that afforded limited flexibility to how staff delivered services and how service users engaged with the service were key barriers to implementation. Policy and procedure change that promoted flexibility in care protocols and offered service users more choice and control over their care were noted as facilitating factors across studies [33, 37, 39, 42, 44, 45, 48, 49].

#### Characteristics of individuals

Characteristics of individuals were reported as barriers and facilitating factors for implementation in nine studies. In all cases, this focussed on staff resistance to change as a barrier [23, 25, 27, 30, 40, 44, 45, 48, 50] and staff openness as a facilitating factor for implementation success [27, 29, 50].

#### Process

All but one of the included studies noted barriers and facilitating factors related to the process of implementation. Most studies identified design and delivery elements of their staff training program as promoting or limiting implementation success. For example, delivery of training to a variety of staff at all levels of the organisation, a flexible format that could be tailored according to needs, practical training elements (e.g. role plays), onsite delivery, ongoing trainings and availability of resources, (as opposed to a once-off session), embedding training into new employee orientation, and making training compulsory were identified as facilitating factors across several studies [25, 29, 30, 46–48, 50–53]. Provision of ongoing modelling, mentoring, and expert consultation promoted uptake and practice change [25, 29, 35–37, 40–42, 47, 48, 34]. Three studies noted that while staff knowledge and confidence in delivering TIC improved, these staff noted a lack of skills training and process changes to actually implement TIC within their organisation [33, 35, 48].

Several studies noted that including service users in implementation efforts promoted implementation success. Relevant strategies included involving a service user as a co-facilitator of training programs, service user inclusion in senior leadership positions and/or implementation teams, seeking regular service user feedback, and designing initiatives in collaboration with service users [36, 40, 44, 50, 51, 34]. Huntington et al. [44] noted that resources and flexibility had to be embedded into the initiative to promote service user engagement (e.g. payment for involvement, prioritising service user schedules). In contrast, a lack of engagement of service users was noted as a barrier to implementation in one study [25].

Finally, several studies reported that establishing mechanisms to collect and regularly review data about the uptake and outcomes of the initiative was a key facilitating factor for change [30, 36, 40, 42, 34]. Others noted a lack of data collection and evaluation within their study as a barrier to implementation, particularly sustainability [25, 38, 54].

## Discussion

This systematic review sought to identify and synthesise evidence about barriers and facilitating factors for implementing TIC into healthcare settings. Given the very high community prevalence of psychological trauma exposure (up to 90% across the lifespan) [1], and the important impacts of trauma exposure when receiving healthcare [5, 6], TIC aims to ensure that care services are safe, empowering, collaborative, and restore power to the care recipient [7]. Implementing TIC into healthcare settings usually requires change at the organisation level to ensure that all staff understand the impacts of psychological trauma, and that processes are in place to modify care behaviour to reduce the risk of re-traumatisation [10, 39]. Like other complex interventions, adaptation of the TIC principles is required for implementation in each specific organisational context [56]. Identifying factors that influence implementation success across initiatives can help to inform the selection of implementation strategies and planning.

Results of this review demonstrate that factors related to the inner organisational setting and process of implementation are most often reported as influencers of TIC implementation success. Implementation was promoted where organisation leadership were highly engaged and committed to TIC and where sufficient resources were allocated to making change in practice. These facilitating factors are commonly reported as essential in efforts to implement innovation in healthcare [57–59], and staff who report having inadequate time for change (whether this is real or perceived) are less likely to implement innovation [58]. Strategies that build innovation into existing processes and procedures are most likely to be sustained and become the ‘normal’ thing to do [60]. For example, in a TIC initiative, this may mean building debriefing into existing case conferencing processes or adding screening items to existing procedures. Several studies included in this review noted that the addition of new processes on top of the existing workload was difficult to facilitate particularly where these processes did not fit into standard consultation times (e.g. 29).

Training staff about psychological trauma is also an essential step in the delivery of TIC particularly in health settings where mental health is not the primary focus of treatment [61]. The mental health literacy of the workforce in these settings can be low, especially where there are limited mental health clinicians on staff [6, 10, 11]. In our review, implementation strategies related to education and training were more comprehensive and multicomponent in non-mental health settings (e.g. primary care, maternal health) than in mental health settings where mental health literacy was high. Our review demonstrates that training efforts are more likely to lead to TIC implementation where they are targeted to staff across all levels of an organisation, with a flexible delivery format, and delivered on an ongoing basis rather than once-off. Training must also be compulsory, as voluntary trainings are unlikely to be well attended [50, 51]. The ongoing availability of experts and mentors (also known as *change agents*) was also an important facilitating factor across studies, consistent with evidence that regular, individualised follow-up is an integral component of success in quality improvement efforts [62]. This may be particularly important for TIC as service users may have complex needs as they contend with the physical, mental, and socio-economic sequelae of their experiences [7].

The process of implementing TIC is promoted where both staff and service users are engaged in both designing the implementation strategy and monitoring its ongoing progress. The value of engaging service users in the co-design of quality improvement initiatives is increasingly recognised and is mutually beneficial for both the service provider and the service user [63]. In the case of TIC, service users can provide nuanced insights about how services can be delivered flexibly and prioritise the needs and preferences of the care user [44]. Flexibility in organisational policy and procedure was a key facilitating factor for implementation across the included studies [33, 37, 39, 42, 44, 45, 48, 49]. In addition, several studies included in this review described the value of infrastructure to collect and monitor data in initiatives to implement TIC. Data collection and monitoring facilitates ongoing review of resource allocation to strategies that are most effective and promotes staff engagement and buy-in [64].

Factors related to the characteristics of the intervention (TIC) and the individuals within the service generally focused on the sense of relevance of TIC for the service and service users, as well as staff openness to change. This is consistent with evidence that any intervention perceived by staff as not useful, not applicable to their clients, or not harmonious with their current practice is difficult to implement in practice [58]. Efforts to build ‘buy-in’ among staff are a crucial element of the knowledge-to-action pipeline [65]. Identifying and upskilling key opinion leaders and advocates for the intervention among the staff (including frontline staff who are well regarded among their peers), aligning the intervention with existing organisational policies and procedures, and creating incentives for use that are meaningful to the staff are key strategies to build staff buy-in [66]. Using participatory co-design methods together with staff to design implementation strategies can help to increase openness to change [67]. These were identified as facilitating factors in studies included in this review [32, 42, 34]. Failure to build staff buy-in can result in low staff morale and staff turnover [66]. Strategies should ensure that staff recognise the prevalence of trauma exposure among their clients and the impacts of these experiences when receiving care, and demonstrate how implementing TIC can support progress toward organisational goals (e.g. reducing responsive behaviour, need for seclusion and restraint, and staff and patient injury) [68].

### Strengths and limitations

Key strengths of most of the identified studies included detailed information about the setting in which implementation occurred and examination of how these factors influenced implementation outcomes. The use of mixed-methods in 15 studies allowed an in-depth triangulation of data [27, 29, 30, 35, 36, 38, 39, 41–43, 46, 52–54, 34]. However, limitations included that most of the included studies were case studies describing a discrete implementation site or region, without a control condition. This limits comparability. In addition, eight studies reported the reflections of the authors, rather than the collection and analysis of empirical data [27, 32, 36, 38, 40, 42, 44, 45, 34]. The results of these studies should therefore be interpreted with caution. None of the included studies explicitly compared the effectiveness of different implementation strategies to each other, and this would be helpful in future work to guide strategy selection and design. Finally, very few studies included a critical analysis of the author’s own role in the implementation and presentation of results. Given that authors were commonly actively involved in implementation, their underlying assumptions, beliefs and experiences are likely to have influenced data collection, analysis and reporting. Future efforts to reduce bias might include the use of external evaluation teams or the inclusion of reflexivity statements in analysis and reporting [69].

Strengths of this review include our broad search strategy that captured initiatives to implement TIC across countries and healthcare settings. Our synthesis generated common themes across diverse initiatives and mapped them to an existing framework to maximise interpretability. Limitations of this review include that we excluded any papers not published in English and this may limit the generalisability of the results. The lack of an operational definition of TIC and the breadth of interventions delivered at the system or organisation level in the included studies may also limit comparability and the conclusions that can be drawn from the results. However, core elements of the implementation strategies were common across studies (e.g. staff training, routine screening) promoting comparability. Future reviews may be helpful for synthesising common implementation strategies used for TIC in more depth. Our coding and mapping to the CFIR framework may have been influenced by subjectivity, though our use of multiple coders and multiple rounds of coding reduces this risk. In addition, we did not assess the relative strength of each influencing factor. That is, factors that were reported less often across studies may nonetheless have a more powerful influence on implementation. The exclusion of an evaluation of relative strength of the influencing factors was primarily determined by few of the included studies providing such an analysis. Future studies that examine the strength of influence of each factor on implementation outcomes will be helpful for filling this research gap.

## Conclusion

There have been recent calls to implement TIC as a universal model of care across healthcare [68], aged care [6], and social care services [9] in recognition of the major impacts of trauma exposure in the receipt of care and the potential harm to care recipients and providers that can result from inappropriate care. However, implementing TIC usually requires a complex organisational change process including both staff behaviour change and organisational policy and procedure change to facilitate staff change [10]. This review identifies key factors that should be targeted to promote TIC implementation, including interagency collaboration, staff and leadership buy-in, aligning implementation strategies with existing policies and procedures, allocation of adequate human and financial resources, flexibility in organisational policies and procedures, ongoing and tailored training, participatory co-design, and the collection and monitoring of data. Continued research will be helpful for characterising what TIC looks like when it is delivered well, and providing validated frameworks to promote organisational uptake for the benefit of trauma survivors.

## Supplementary Information

**Additional file 1. **PRISMA Checklist; Search strategy; PRISMA diagram; Results of quality appraisal; Mixed-methods appraisal tool questions. The tile contains tables that provide additional information about the systematic review process. This includes the search strategy, PRISMA checklist and diagram, and tables pertaining to the quality appraisal undertaken.

## Data Availability

The datasets used and/or analysed during the current study are available from the corresponding author on reasonable request.
