# Supplementary material for: Barriers and enablers for the implementation of trauma-informed care in healthcare settings: a systematic review
Source: Implement Sci Commun. 2023 May 5;4:49. doi: 10.1186/s43058-023-00428-0 (PMC10161455; doi:10.1186/s43058-023-00428-0)
Supplement: Supplementary file 1 — Additional file 1. PRISMA Checklist; Search strategy; PRISMA diagram; Results of quality appraisal; Mixed-methods appraisal tool questions. The tile contains tables that provide additional information about the systematic review process. This includes the search strategy, PRISMA checklist and diagram, and tables pertaining to the quality appraisal undertaken. [file 43058_2023_428_MOESM1_ESM.docx]

**SUPPORTING INFORmation**

Table S1. PRISMA Checklist

| **Section and Topic** | | **Item #** | | **Checklist item** | | **Location where item**  **is reported** | |
| --- | --- | --- | --- | --- | --- | --- | --- |
| **TITLE** | | | | | |  | |
| Title | | 1 | | Identify the report as a systematic review.  *Barriers and enablers for the implementation of trauma-informed care in healthcare settings: A systematic review* | | Title | |
| **ABSTRACT** | | | | | |  | |
| Abstract | | 2 | | See the PRISMA 2020 for Abstracts checklist.  *Please see abstract paragraphs 1-4.* | | Abstract,  paragraphs 1-4 | |
| **INTRODUCTION** | | | | | |  | |
| Rationale | | 3 | | Describe the rationale for the review in the context of existing knowledge.  *Factors that promote or impede implementation of TIC are not yet well characterised and understood (15). Understanding contextual, organisational, and implementation-specific factors that promote the uptake and effectiveness of TIC will help to guide future efforts to implement TIC more widely*. | | Background,  paragraph 4 | |
| Objectives | | 4 | | Provide an explicit statement of the objective(s) or question(s) the review addresses.  *The aim of this review was to systematically identify and synthesise evidence regarding factors that promote or reduce the effectiveness and/or implementation of TIC in healthcare settings.* | | Background  paragraph 5 | |
| **METHODS** | | | | | |  | |
| Eligibility criteria | | 5 | | Specify the inclusion and exclusion criteria for the review and how studies were grouped for the syntheses.  *We included studies published since the year 2000 and in English, reporting original research or evaluation, and that reporting barriers and/or facilitating factors for the effectiveness or implementation of a TIC in a health care setting. Studies published prior to 2000 were excluded because the TIC framework was not well-defined in research before this time (13).* | | Methods,  paragraphs 3-5 | |
| Information sources | | 6 | | Specify all databases, registers, websites, organisations, reference lists and other sources searched or consulted to identify studies. Specify the date when each source was last searched or consulted.  *We conducted a search of Scopus, MEDLINE, Proquest, and PsycINFO for English language studies published from January 2000 to April 2021* | | Methods,  paragraph 2 | |
| Search strategy | | 7 | | Present the full search strategies for all databases, registers and websites, including any filters and limits used. | | Table S2 | |
| Selection process | | 8 | | Specify the methods used to decide whether a study met the inclusion criteria of the review, including how many reviewers screened each record and each report retrieved, whether they worked independently, and if applicable, details of automation tools used in the process.  *One reviewer (YH) screened all titles and removed irrelevant papers. Abstracts and full texts were screened for eligibility by two reviewers (YH and MC) using an eligibility checklist based on the criteria described above. Disagreements about inclusion were resolved via discussion between the reviewers, and a third reviewer (LC) was consulted where consensus could not be reached*. | | Methods,  paragraph 6 | |
| Data collection process | | 9 | | Specify the methods used to collect data from reports, including how many reviewers collected data from each report, whether they worked independently, any processes for obtaining or confirming data from study investigators, and if applicable, details of automation tools used in the process.  *Data extraction was conducted by one reviewer (YH) using a data extraction spreadsheet that was piloted with five studies before being finalised and being used with the remaining studies. The accuracy of data extraction was validated by a second reviewer.* | | Methods,  paragraph 6 | |
| Data items | | 10a | | List and define all outcomes for which data were sought. Specify whether all results that were compatible with each outcome domain in each study were sought (e.g. for all measures, time points, analyses), and if not, the methods used to decide which results to collect.  *Data synthesis followed a two-step process modelled on the method of a recent review of barriers and facilitating factors for person-centred care in long-term care setting*s | | N/A | |
|  |  | 10b | | List and define all other variables for which data were sought (e.g. participant and intervention characteristics, funding sources). Describe any assumptions made about any missing or unclear information.  *Extracted data included the study first author and year of publication, design, setting, population, number of sites, initiative elements (e.g. implementation strategies), outcome data type (e.g. qualitative, quantitative, mixed), evaluation method, outcomes reported, barriers identified, and facilitating factors identified.* | | Methods,  paragraph 6 | |
| Study risk of bias assessment | | 11 | | Specify the methods used to assess risk of bias in the included studies, including details of the tool(s) used, how many reviewers assessed each study and whether they worked independently, and if applicable, details of automation tools used in the process.  *The Mixed Methods Appraisal Tool (MMAT) Checklist was used to assess the quality of each included study. The validity, robustness, and applicability of each included study was appraised by two team members (YH and BW-H) independently and in duplicate (21).* | | Methods,  paragraph 7 | |
| Effect measures | | 12 | | Specify for each outcome the effect measure(s) (e.g. risk ratio, mean difference) used in the synthesis or presentation of results. | | N/A | |
| Synthesis methods | | 13a | | Describe the processes used to decide which studies were eligible for each synthesis (e.g. tabulating the study intervention characteristics and comparing against the planned groups for each synthesis (item #5)).  *Data synthesis followed a two-step process modelled on the method of a recent review of barriers and facilitating factors for person-centred care in long-term care settings (22). First, two authors (YH and MC) independently used a thematic analysis approach to group barriers and facilitating factors into recurrent themes (e.g. lack of collaboration, time constraints). Themes were consolidated on discussion and a second independent round of coding was conducted by both reviewers with new emerging themes added to the codebook iteratively. A final, third round of coding was conducted by one reviewer for refinement (MC).* | | Methods,  paragraph 8 | |
|  |  | 13b | | Describe any methods required to prepare the data for presentation or synthesis, such as handling of missing summary statistics, or data conversions. | | N/A | |
|  |  | 13c | | Describe any methods used to tabulate or visually display results of individual studies and syntheses. | | N/A | |
|  |  | 13d | | Describe any methods used to synthesize results and provide a rationale for the choice(s). If meta-analysis was performed, describe the model(s), method(s) to identify the presence and extent of statistical heterogeneity, and software package(s) used.  *In the second step of synthesis, the themes were mapped to the Consolidated Framework for Implementation Research (CFIR) by both reviewers (23).* | | Methods,  paragraph 8 | |
|  |  | 13e | | Describe any methods used to explore possible causes of heterogeneity among study results (e.g. subgroup analysis, meta-regression). | | N/A | |
|  |  | 13f | | Describe any sensitivity analyses conducted to assess robustness of the synthesized results. | | N/A | |
| Reporting bias assessment | | 14 | | Describe any methods used to assess risk of bias due to missing results in a synthesis (arising from reporting biases). | | N/A | |
| Certainty assessment | | 15 | | Describe any methods used to assess certainty (or confidence) in the body of evidence for an outcome.  *The Mixed Methods Appraisal Tool (MMAT) Checklist was used to assess the quality of each included study. The validity, robustness, and applicability of each included study was appraised by two team members (YH and BW-H) independently and in duplicate (21).* | | Methods,  paragraph 7 | |
| **RESULTS** | | | | | |  | |
| Study selection | | 16a | | Describe the results of the search and selection process, from the number of records identified in the search to the number of studies included in the review, ideally using a flow diagram.  *The initial search identified 3051 original results, of which 170 were retrieved in full text and screened against the review inclusion and exclusion criteria. Most exclusions were due to the implementation occurring in a non-health related care setting. A total of 27 studies were included, reported across 28 publications* | | Results,  paragraph 1; Figure S1 | |
|  |  | 16b | | Cite studies that might appear to meet the inclusion criteria, but which were excluded, and explain why they were excluded. | | N/A | |
| Study characteristics | | 17 | | Cite each included study and present its characteristics.  *Please see Table 1* | | Table 1 | |
| Risk of bias in studies | | 18 | | Present assessments of risk of bias for each included study.  *Please see Table S4.* | | Table S4 | |
| Results of individual studies | | 19 | | For all outcomes, present, for each study: (a) summary statistics for each group (where appropriate) and (b) an effect estimate and its precision (e.g. confidence/credible interval), ideally using structured tables or plots. | | N/A | |
| Results of syntheses | | 20a | | For each synthesis, briefly summarise the characteristics and risk of bias among contributing studies. | | N/A | |
|  |  | 20b | | Present results of all statistical syntheses conducted. If meta-analysis was done, present for each the summary estimate and its precision (e.g. confidence/credible interval) and measures of statistical heterogeneity. If comparing groups, describe the direction of the effect. | | N/A | |
|  |  | 20c | | Present results of all investigations of possible causes of heterogeneity among study results. | | N/A | |
|  |  | 20d | | Present results of all sensitivity analyses conducted to assess the robustness of the synthesized results. | | N/A | |
| Reporting biases | | 21 | | Present assessments of risk of bias due to missing results (arising from reporting biases) for each synthesis assessed. | | N/A | |
| Certainty of evidence | | 22 | | Present assessments of certainty (or confidence) in the body of evidence for each outcome assessed.  *Please see Table S4.* | | Table S4 | |
| **DISCUSSION** | | | | | |  | |
| Discussion | | 23a | | Provide a general interpretation of the results in the context of other evidence.  *Please see discussion paragraphs 1-5.* | | Discussion, paragraph 1-5 | |
|  |  | 23b | | Discuss any limitations of the evidence included in the review.  *Limitations included that most of the included studies were case studies describing a discrete implementation site or region, without a control condition. This limits comparability. In addition, eight studies reported the reflections of the authors, rather than the collection and analysis of empirical data (28,30–32,34,36,37,42,43). The results of these studies should therefore be interpreted with caution. Finally, very few studies included a critical analysis of the author’s own role in the implementation and presentation of results.* | | Discussion, paragraph 6 | |
|  |  | 23c | | Discuss any limitations of the review processes used.  *Limitations of this review include that we excluded any papers not published in English and this may limit the generalisability of the results. Our coding and mapping to the CFIR framework may have been influenced by subjectivity, though our use of multiple coders and multiple rounds of coding reduces this risk. In addition, we did not assess the relative strength of each influencing factor. That is, factors that were reported less often across studies may nonetheless have a more powerful influence on implementation* | | Discussion, paragraph 7 | |
|  |  | 23d | | Discuss implications of the results for practice, policy, and future research.  *Please see discussion paragraphs 1-5 and 8* | | Discussion, paragraph 1-5, 8 | |
| **OTHER INFORMATION** | | | | | |  | |
| Registration and protocol | | 24a | | Provide registration information for the review, including register name and registration number, or state that the review was not registered.  *The review protocol was registered on the PROSPERO database (CRD42021242891).* | | Methods,  paragraph 1 | |
|  |  | 24b | | Indicate where the review protocol can be accessed, or state that a protocol was not prepared.  *The review protocol was registered on the PROSPERO database (CRD42021242891).* | | N/A | |
|  |  | 24c | | Describe and explain any amendments to information provided at registration or in the protocol. | | N/A | |
| Support | | 25 | | Describe sources of financial or non-financial support for the review, and the role of the funders or sponsors in the review.  *This study is funded by the South Australian Hospital Research Foundation and the Australian Government Medical Research Future Fund. AA is supported by an Early Career Fellowship from the South Australian Hospital Research Foundation and a National Health and Medical Research Council Medical Research Future Fund Emerging Leadership Investigator Grant. BB is supported by an Australian Research Council Discovery Early Career Researcher Award. CC is supported by a NHMRC Boosting Dementia Research Leadership Fellowship. The funders had no role in study design, data collection and analysis, decision to publish, or preparation of the manuscript.* | | Funding statement | |
| Competing interests | | 26 | | Declare any competing interests of review authors.  *No competing interests to declare* | | Competing interests statement | |
| Availability of data, code and other materials | | 27 | | Report which of the following are publicly available and where they can be found: template data collection forms; data extracted from included studies; data used for all analyses; analytic code; any other materials used in the review. | | Data availability statement | |

Table S2. Search strategy applied to PsycINFO.

| **Intervention** | Trauma-informed care | (ab((trauma-informed OR trauma-sensitive OR trauma-focus* OR trauma-reduct* OR trauma-awar* OR trauma-support*) NEAR/4 (care OR practi*e OR approach* OR program* OR support* OR service* OR system* OR intervention)) OR ti ((trauma-informed OR trauma-sensitive OR trauma-focus* OR trauma-reduct* OR trauma-awar* OR trauma-support*) NEAR/4 (care OR practi*e OR approach* OR program* OR support* OR service* OR system* OR intervention))) AND (ab((trauma-informed OR trauma-sensitive OR trauma-focus* OR trauma-reduct* OR trauma-awar* OR trauma-support*) NEAR/4 (care OR practi*e OR approach* OR program* OR support* OR service* OR system* OR intervention)) OR ti ((trauma-informed OR trauma-sensitive OR trauma-focus* OR trauma-reduct* OR trauma-awar* OR trauma-support*) NEAR/4 (care OR practi*e OR approach* OR program* OR support* OR service* OR system* OR intervention))) |
| --- | --- | --- |
| **Setting** | Health service | ab((organi*ation* OR (school* OR educat* OR hospital* OR servic* OR facilit* OR compan* OR firm* OR operation* OR corporat* OR institut* OR establishment* OR body OR bodies OR agenc* OR system* OR group* OR resident* OR site* OR setting*)) OR (organi*ation* n/10 (school* OR educat* OR hospital* OR servic* OR facilit* OR compan* OR firm* OR operation* OR corporat* OR institut* OR establishment* OR body OR bodies OR agenc* OR system* OR group* OR resident* OR site* OR setting*)) OR (workforce* or staff or "human resourc*")) OR ti((organi*ation* OR (school* OR educat* OR hospital* OR servic* OR facilit* OR compan* OR firm* OR operation* OR corporat* OR institut* OR establishment* OR body OR bodies OR agenc* OR system* OR group* OR resident* OR site* OR setting*)) OR (organi*ation* n/10 (school* OR educat* OR hospital* OR servic* OR facilit* OR compan* OR firm* OR operation* OR corporat* OR institut* OR establishment* OR body OR bodies OR agenc* OR system* OR group* OR resident* OR site* OR setting*)) OR (workforce* or staff or "human resourc*")) |
| **Outcome** | Barriers and facilitating factors for implementation | ab((barrier* OR block* OR prevent* OR obstacle* OR difficult* OR obstruct* OR barricad* OR challeng* OR lesson*) OR (adoption or acceptability or feasibility or sustain* or maintain*or fidelity or follow through) OR (facilitat* OR enable* OR promot* OR contribut* OR advocat* OR success*OR support*)) OR ti((barrier* OR block* OR prevent* OR obstacle* OR difficult* OR obstruct* OR barricad* OR challeng* OR lesson*) OR (adoption or acceptability or feasibility or sustain* or maintain*or fidelity or follow through) OR (facilitat* OR enable* OR promot* OR contribut* OR advocat* OR success*OR support*)) |
| **Year** | January 2000 -current | Limit to January 2000-current |

Records sought for retrieval

(*n*=171)

Reports not retrieved (*n*=1)

Records assessed for eligibility

(*n*=170)

Reports excluded:

- Not health setting (*n*=91)
- Not organisational intervention (*n*=23)
- Barriers or facilitating factors not reported (*n*=19)
- Not an empirical study (*n*=8)
- No implementation of trauma-informed care (*n*=1)

Reports assessed for eligibility (*n*=2)

Reports excluded:

- No implementation of trauma-informed care (*n*=2) (n = 1)

Studies included in review

(*n*=27)32)

Reports included in review (*n*=28)

**Screening**

**Included**

Record title and abstracts screened (*n*=3051)

Records excluded

(*n*=2880)

Total records identified from database search

(*n*=5087)

Duplicate records removed (*n*=2036)

Records identified from:

- Citation searching (*n*=18)
- Grey literature searching (*n*=15)

**Identification of studies via databases and registers**

**Identification of studies via other methods**

**Identification**

Reports sought for retrieval

(*n*=2)

Figure S1. PRISMA diagram of included studies.

Table S3. Mixed-Methods Appraisal Tool questions.

| **Category of study designs** | **Methodological quality criteria** |
| --- | --- |
|  |  |
| Screening questions  (for all types) | S1. Are there clear research questions? |
|  | S2. Do the collected data allow to address the research questions? |
| 1. Qualitative | 1.1. Is the qualitative approach appropriate to answer the research question? |
|  | 1.2. Are the qualitative data collection methods adequate to address the research question? |
|  | 1.3. Are the findings adequately derived from the data? |
|  | 1.4. Is the interpretation of results sufficiently substantiated by data? |
|  | 1.5. Is there coherence between qualitative data sources, collection, analysis and interpretation? |
| 2. Quantitative  randomized controlled trials | 2.1. Is randomization appropriately performed? |
|  | 2.2. Are the groups comparable at baseline? |
|  | 2.3. Are there complete outcome data? |
|  | 2.4. Are outcome assessors blinded to the intervention provided? |
|  | 2.5 Did the participants adhere to the assigned intervention? |
| 3. Quantitative non-  randomized | 3.1. Are the participants representative of the target population? |
|  | 3.2. Are measurements appropriate regarding both the outcome and intervention (or exposure)? |
|  | 3.3. Are there complete outcome data? |
|  | 3.4. Are the confounders accounted for in the design and analysis? |
|  | 3.5. During the study period, is the intervention administered (or exposure occurred) as intended? |
| 4. Quantitative  descriptive | 4.1. Is the sampling strategy relevant to address the research question? |
|  | 4.2. Is the sample representative of the target population? |
|  | 4.3. Are the measurements appropriate? |
|  | 4.4. Is the risk of nonresponse bias low? |
|  | 4.5. Is the statistical analysis appropriate to answer the research question? |
| 5. Mixed methods | 5.1. Is there an adequate rationale for using a mixed methods design to address the research question? |
|  | 5.2. Are the different components of the study effectively integrated to answer the research question? |
|  | 5.3. Are the outputs of the integration of qualitative and quantitative components adequately interpreted? |
|  | 5.4. Are divergences and inconsistencies between quantitative and qualitative results adequately addressed? |
|  | 5.5. Do the different components of the study adhere to the quality criteria of each tradition of the methods involved? |

Table S4. Results of Quality Appraisal using Mixed-Methods Appraisal Tool (see Table S4 for questions).

| **Study first author (Year)** | **Appraisal Item** | | | | | | | | | | | | | | | | | | | | | |
| --- | --- | --- | --- | --- | --- | --- | --- | --- | --- | --- | --- | --- | --- | --- | --- | --- | --- | --- | --- | --- | --- | --- |
|  | **S1** | **S2** | **1.1** | **1.2** | **1.3** | **1.4** | **1.5** | **3.1** | **3.2** | **3.3** | **3.4** | **3.5** | **4.1** | **4.2** | **4.3** | **4.4** | **4.5** | **5.1** | **5.2** | **5.3** | **5.4** | **5.5** |
| Qualitative only | | | | | | | | | | | | | | | | | | | | | | |
| Amaro (2005) | Y | Y | Y | Y | CT | CT | N | - | - | - | - | - | - | - | - | - | - | - | - | - | - | - |
| Chandler (2008) | Y | Y | Y | Y | Y | Y | Y | - | - | - | - | - | - | - | - | - | - | - | - | - | - | - |
| Huntington (2005) & Moses (2003) | Y | Y | Y | CT | CT | CT | CT | - | - | - | - | - | - | - | - | - | - | - | - | - | - | - |
| Koury (2017) | Y | Y | CT | CT | CT | CT | N | - | - | - | - | - | - | - | - | - | - | - | - | - | - | - |
| Levine (2021) | Y | Y | Y | Y | Y | Y | Y | - | - | - | - | - | - | - | - | - | - | - | - | - | - | - |
| Mantler (2018) | Y | Y | Y | Y | Y | Y | Y | - | - | - | - | - | - | - | - | - | - | - | - | - | - | - |
| Loomis (2019) | Y | Y | Y | CT | CT | N | N | - | - | - | - | - | - | - | - | - | - | - | - | - | - | - |
| McEvedy (2017) | Y | Y | Y | Y | Y | Y | Y | - | - | - | - | - | - | - | - | - | - | - | - | - | - | - |
| Quantitative only | | | | | | | | | | | | | | | | | | | | | | |
| Beidas (2016) | Y | Y | - | - | - | - | - | Y | Y | N | CT | Y | - | - | - | - | - | - | - | - | - | - |
| Dike (2020) | Y | Y | - | - | - | - | - | Y | Y | Y | N | Y | - | - | - | - | - | - | - | - | - | - |
| Korchmaros (2021) | Y | Y | - | - | - | - | - | Y | Y | Y | N | Y | - | - | - | - | - | - | - | - | - | - |
| McNamara (2021) | Y | Y | - | - | - | - | - | CT | Y | N | N | Y | - | - | - | - | - | - | - | - | - | - |
| Mixed methods | | | | | | | | | | | | | | | | | | | | | | |
| Azeem (2015) | Y | Y | Y | N | N | N | CT | - | - | - | - | - | Y | Y | Y | CT | Y | N | N | N | Y | N |
| Bartlett (2016) | Y | Y | Y | Y | Y | Y | Y | Y | Y | CT | Y | Y | - | - | - | - | - | Y | Y | Y | Y | Y |
| Caldwell (2014) | Y | Y | Y | Y | CT | N | N | Y | CT | CT | CT | Y | - | - | - | - | - | N | N | CT | N | N |
| Conover (2015) | CT | CT | CT | CT | CT | N | N | Y | Y | Y | CT | Y | - | - | - | - | - | N | N | N | N | CT |
| Damian (2017) | Y | Y | Y | Y | Y | Y | Y | Y | Y | CT | Y | Y | - | - | - | - | - | Y | Y | Y | Y | Y |
| Dorr (2019) | Y | Y | Y | N | CT | CT | CT | Y | Y | Y | Y | N | - | - | - | - | - | CT | CT | CT | CT | N |
| Dueweke (2019) | Y | Y | Y | Y | Y | Y | Y | Y | Y | Y | Y | Y | - | - | - | - | - | Y | Y | Y | Y | Y |
| Hale (2020) | Y | Y | Y | N | N | N | N | Y | Y | Y | Y | Y | - | - | - | - | - | N | N | N | CT | N |
| Hall (2016) | Y | Y | Y | Y | Y | Y | Y | Y | Y | Y | N | Y | - | - | - | - | - | Y | Y | CT | Y | Y |
| Jee (2020) | Y | Y | Y | Y | Y | Y | Y | Y | Y | N | N | Y | - | - | - | - | - | Y | Y | Y | Y | Y |
| Palfrey (2019) | Y | Y | Y | Y | Y | Y | Y | Y | Y | Y | N | Y | - | - | - | - | - | Y | Y | Y | Y | Y |
| Purbeck (2020) | Y | Y | Y | Y | Y | Y | Y | Y | Y | Y | N | Y | - | - | - | - | - | Y | Y | Y | Y | Y |
| Sala-Hamrick (2021) | Y | Y | Y | Y | CT | Y | CT | Y | CT | CT | N | Y | - | - | - | - | - | Y | N | Y | Y | Y |
| Simonich (2015) | Y | Y | Y | N | N | N | CT | Y | Y | CT | CT | Y | - | - | - | - | - | Y | N | CT | CT | Y |
| Tuck (2017) | Y | Y | Y | Y | Y | N | N | - | - | - | - | - | Y | Y | CT | CT | CT | Y | N | CT | N | CT |

Y = Yes, N= No, CT=Cannot tell, - = Not applicable
